# Skin Health Function of Distilled Soju Byproduct Fermented with *Saccharomyces cerevisiae* MGE 3400

**DOI:** 10.4014/jmb.2510.10038

**Published:** 2026-01-22

**Authors:** Md Asaduzzaman, Ji-Ho Park, Gi-Seong Moon

**Affiliations:** 1 Major in Biotechnology, Korea National University of Transportation, Jeungpyeong 27909, Republic of Korea; 2 Major in IT·Biohealth Convergence, Department of IT·Energy Convergence, Graduate School, Korea National University of Transportation, Chungju 27469, Republic of Korea; 3 4D Convergence Technology Institute, Korea National University of Transportation, Jeungpyeong 27909, Republic of Korea

**Keywords:** Skin health, Distilled soju byproduct, *Saccharomyces cerevisiae*, UV protection, fermentation, Waste valorization, Cosmetic application

## Abstract

The skin serves as a vital barrier against environmental and biological stressors, and its impairment leads to premature aging and various dermatological disorders. The growing demand for natural, sustainable cosmetic ingredients has drawn attention to food industry byproducts as potential bioactive sources. Production of distilled soju generates substantial byproducts that are typically discarded but possess valuable metabolites. This study investigated the skin health–related functions of distilled soju byproducts fermented with the *Saccharomyces cerevisiae* MGE 3400 as a starter culture, which was isolated from a nuruk, compared with a commercial yeast. In vitro assays were performed to evaluate antioxidant activity and antimicrobial activity against the skin pathogens *Candida albicans*, *Staphylococcus aureus*, and *Cutibacterium acnes*, as well as moisturizing-related HAS2 expression, UV-protective effects, and wound-healing properties using the HaCaT cell line. *S. cerevisiae* MGE 3400 fermented distilled soju byproduct showed stronger antioxidant and antimicrobial activities than the control. In addition, they promoted the expression of hyaluronan synthase 2 (HAS2) and insulin-like growth factor 1 (IGF-1) genes, enhanced ultraviolet (UV) damage protection, and accelerated wound closure in keratinocyte HaCaT cells, suggesting an overall improvement in skin-regenerative potential. These findings suggest that *S. cerevisiae* MGE 3400 can enhance the functional properties of distilled soju byproduct, supporting their use as a sustainable source of natural ingredients for cosmetic and dermatological applications.

## Introduction

As the largest organ in the human body, the skin acts as a barrier to protect against a variety of biological and environmental stressors, including physical agents, chemical irritants, pathogenic microorganisms, and ultraviolet radiation [1]. It maintains homeostasis by preventing water loss, regulating temperature, and participating in immune defense [2]. When these processes are disrupted, the skin ages more quickly, the integrity of the barrier is compromised, and the vulnerability to dermatological illnesses increases [3, 4].

In recent years, there has been a growing consumer demand for natural, plant-based, and microorganism-derived ingredients in skin care formulations [5, 6]. The need for sustainable and environmentally friendly cosmetics as well as increasing awareness of the possible negative effects of synthetic chemicals are the main drivers of this change [7]. Natural bioactive compounds such as polyphenols, peptides, and polysaccharides are known for their multifunctional properties, including antioxidant, antimicrobial, anti-inflammatory, moisturizing, and wound-healing effects [8–10]. Importantly, utilizing food industry byproducts as sources of these compounds not only addresses environmental concerns by reducing waste but also offers a cost-effective strategy for developing novel cosmetic and dermatological products [11].

Fermentation has been considered one of the most potent bioprocessing technologies that could enhance the bioactivity of natural materials [12]. Through enzymatic breakdown and microbial metabolism, fermentation can release bound phenolic compounds, generate bioactive peptides, and produce secondary metabolites with improved stability and bioavailability [13]. In the cosmetic industry, fermented ingredients have been shown to exhibit enhanced antioxidant capacity, stronger antimicrobial properties, better skin absorption, and reduced irritation potential compared to their unfermented counterparts [14, 15].

In these days, one of the most popular alcoholic beverages in Korea is distilled soju, for which two steps of fermentation and distillation are included and fermentation process is exactly the same as makgeolli fermentation fermentation process, where *Saccharomyces cerevisiae* plays a key role to produce ethanol as well as modulate key sensory attributes such as aroma, taste, and texture [16]. The selection of the yeast strain is crucial since it affects the production of flavour compounds and other fermentation metabolites in addition to the ethanol yield and fermentation efficiency [17]. In distilled soju production, commercial yeast for bakery is often used because of its low cost, easy growth, and consistent ethanol production [18]. However, these strains may not be ideal for maximizing the nutritional or functional value of fermentation byproducts because they are primarily designed for alcohol fermentation performance [19].

Recent research has shown that byproducts from alcoholic beverage fermentation, such as those generated during shochu and sake production, contain various bioactive compounds such as polyphenols, peptides, and organic acids. These substances have exhibited antioxidant, antimicrobial, and skin-protective activities, suggesting potential applications in health and cosmetic products [20–23]. The production of distilled soju generates substantial amounts of solid and liquid byproducts, which are often discarded or used for low-value applications such as animal feed [24]. These byproducts may contain functional metabolites that can be utilized in the pharmaceutical and cosmetic industries [25, 26]. The sustainability and profitability of soju production could be increased by increasing the amount of these compounds in byproducts, which could then be converted into resources with additional value. One way to achieve this is through the use of specialized yeast strains designed to produce higher levels of desirable metabolites [27].

*S. cerevisiae* MGE 3400 strain was isolated from a nuruk and applied for this study to confirm whether it shows improved skin health functions compared with commercial yeast starter. Therefore, the aim of this study was to compare the skin health–related bioactivities of distilled soju byproducts produced using either commercial yeast or *S. cerevisiae* MGE 3400. Our results suggest that the soju byproduct produced with *S. cerevisiae* MGE 3400 show enhanced skin health–related properties, such as stronger antimicrobial activities against skin pathogens such as *C. albicans*, *S. aureus*, and *C. acnes*, improved skin moisturizing, antioxidant, UV damage protection, and wound-healing potential. By establishing *S. cerevisiae* MGE 3400 as a starter culture that can produce high-quality distilled soju and value-added byproducts, this work provides a scientific basis for its potential commercial application.

## Materials and Methods

### Cell Culture

HaCaT cells were obtained from CLS Cell Lines Services GmbH (Germany) and cultured in DMEM supplemented with 10% fetal bovine serum (FBS) and 1% penicillin-streptomycin. Cells were maintained at 37°C in a humidified incubator with 5% CO_2_ and subcultured upon reaching confluency for use in subsequent experiments.

### Preparation of Distilled Soju Byproduct (DSBP)

DSBP was prepared using two different yeast strains: a commercial yeast (La Parisienne, DB ingredients, UK) and *S. cerevisiae* MGE 3400, which was isolated from a homemade nuruk and deposited in the Korean Collection for Type Culture (KCTC 16217BP). These DSBP samples were prepared and provided by Danong Bio Co. (Republic of Korea). The DSBP samples were mixed thoroughly and centrifuged at 15,000 rpm for 10 min. The supernatant was collected, centrifuged again under the same conditions, and filtered through a 0.45 μm PVDF membrane filter (GVS North America, USA). The resulting filtrate was freeze-dried to obtain a powdered form and stored at −20°C until use. For experiments, the freeze-dried powder was dissolved in sterilized 1× PBS to prepare a 100,000 ppm stock solution, which was then diluted to 5,000, 10,000, 25,000, 50,000, or 75,000 ppm as required.

### Antioxidant Activity Determination

The antioxidant activity of the DSBP was measured using the 2,2-diphenyl-1-picrylhydrazyl (DPPH) radical scavenging assay. Freeze-dried DSBP powder was dissolved in ultrapure water (3DW) to a concentration of 100,000 ppm. Sodium L-ascorbate (Sigma-Aldrich, USA) was used as a reference antioxidant control and prepared at 0-200 ppm. A 2 mM DPPH solution was prepared in methanol (Honeywell Burdick & Jackson, USA). For the assay, 200 μl of 2 mM DPPH solution was mixed with 10 μl of the prepared sample or standard in a 96-well plate. The reaction mixtures were incubated at room temperature for 20 min in the dark, and absorbance was measured at 517 nm. Antioxidant activity (%) was calculated relative to 100 ppm ascorbate.

### Determination of Total Phenolic Content

The total phenolic content (TPC) of the DSBP was determined using the Folin–Ciocalteu method. Freeze-dried DSBP powder was dissolved in 3DW to a concentration of 10,000 ppm. For reaction, 1 ml of the sample was mixed with 1 ml of 2 M Folin–Ciocalteu reagent (Sigma-Aldrich) and allowed to stand for 5 min at room temperature. Subsequently, 400 μl of 7.5% (w/v) sodium carbonate solution was added, the mixture was vortexed, and incubated at 40°C for 30 min in the dark. Following incubation, the samples were centrifuged at 10,000 rpm for 3 min, and 200 μl of the supernatant was transferred to a 96-well plate. Absorbance was measured at 765 nm. Gallic acid (Sigma-Aldrich) was used as the standard, and total phenolic content was expressed as ppm gallic acid equivalents (GAE) per ml of DSBP solution.

### Antimicrobial Activity

The antimicrobial effect of DSBP was evaluated against *C. acnes* KCTC 3314, *S. aureus* KCTC 3881, and *C. albicans* KCTC 7270. Reinforced Clostridial Medium (RCM; BD, USA) was used to culture *C. acnes* under anaerobic conditions, whereas *S. aureus* and *C. albicans* were cultured aerobically in MRS and YPD broth (BD), respectively. Cultures were incubated for 12 h, subcultured two to three times, and then centrifuged at 12,000 rpm for 3 min. The pellets were washed twice with 3DW. Each 1 ml sample containing either PBS (control) or DSBP (25,000 or 50,000 ppm) was inoculated with 10 μl (1% v/v) of the washed microbial suspension. Samples were incubated under appropriate conditions, and colony-forming units (CFU/ml) were measured at 0, 6, and 12 h.

### HaCaT Cell Viability Assay

Cell viability was assessed using the MTT assay. HaCaT keratinocytes were seeded in 96-well plates (1 × 10^4^ cells/well) and treated with distilled soju byproducts fermented with commercial yeast or *S. cerevisiae* MGE 3400 (100–5,000 ppm) for 24, or 48 h. After treatment, 10 μl of MTT solution (5 mg/ml, prepared in 0.22-μm–filtered 3DW) was added to each well and incubated for 2 h at 37°C in the dark. The medium was removed, 100 μl of DMSO was added, and the plates were shaken at 150 rpm for 30 min. Absorbance was measured at 570 nm using a microplate reader (BioTek Synergy HTX, USA).

### Evaluation of HAS2 Expression Related to Moisturizing Function

HaCaT cells were prepared as described in the Cell Culture section. Briefly, cells were seeded into 6-well plates at 8.0 × 10^4^ cells/ml (2 ml per well) and incubated for 24 h to allow attachment. The medium was then replaced with serum-free DMEM, and the cells were starved for 24 h. After starvation, cells were treated with DSBP at final concentrations of 500, 1,000, 2,500, and 5,000 ppm for 12 h, while control cells received 10% PBS in DMEM. HAS2 protein expression related to moisturizing activity was analyzed by western blotting.

### Western Blot Assay

After treatment, cells were washed twice with 1× PBS and lysed with RIPA buffer (Biosesang, Republic of Korea) containing protease inhibitors (1× final concentration). Cell lysates were collected using a cell scraper and centrifuged at 13,000 rpm for 10 min at 4°C. The supernatants were collected and stored at −20°C until analysis. Protein concentrations were determined using the Pierce BCA Protein Assay Kit (Thermo Fisher Scientific, USA). Equal amounts of protein were separated by 12% SDS-PAGE and transferred to PVDF membranes (Bio-Rad) at 100 V for 120 min. Membranes were blocked with 5% skim milk in TBST for 1 h at room temperature, then incubated overnight at 4°C with primary antibodies against HAS2 and β-actin (Santa Cruz Biotechnology, USA) diluted in 5% BSA in TBST. After three washes with TBST, membranes were incubated with an HRP-conjugated secondary antibody (Gendepot, USA) for 2–3 h at room temperature. Bands were visualized using ECL reagent (iNtRON Biotechnology, Republic of Korea) and detected with a ChemiDoc imaging system (Bio-Rad). Band intensities were quantified using ImageJ software and normalized to β-actin.

### Quantitative Real-Time PCR (qRT-PCR)

Total RNA was extracted from HaCaT cells treated with distilled soju byproduct (DSBP) using the RNeasy Mini Kit (Qiagen, Germany) according to the manufacturer’s instructions. The purified RNA was eluted in 50 μl of nuclease-free water, and its concentration and purity were determined using a NanoDrop spectrophotometer (Microdigital, Republic of Korea). For gene expression analysis, qRT-PCR was performed using the QuantStudio 5 Real-Time PCR System (Thermo Fisher Scientific, USA). qRT-PCR reactions were prepared using the HS One-step RT-qPCR 2X Master Mix (SYBR Green, low ROX) (Elpis, Republic of Korea). Each 20 μl reaction contained 10 μl of master mix, 1 μl of each forward and reverse primer (10 pmol/μl), 80 ng/μl of template RNA, and 7 μl of nuclease-free water to reach the final volume. Thermal cycling was performed under the following conditions: reverse transcription at 50°C for 10 min, initial denaturation at 95°C for 3 min, followed by 45 cycles of denaturation at 95°C for 15 sec and annealing/extension at 60°C for 60 sec. The specific primers used were: IGF-1 (Forward: 5’-CTAACACTC AGCAGGTCTTCCA-3’; Reverse: 5’-GGTGCTCCTCCTCAGATCCT-3’) and β-actin (Forward: 5’-CACCATGTACGTTGCTATCC-3’; Reverse: 5’-CGATCCACACGGAGTACTTG-3’). Each sample was analyzed in triplicate to ensure reproducibility, and β-actin was used as an internal control to normalize gene expression levels. The relative changes in gene expression were calculated using the 2^^-ΔΔCt^ method, and results were presented as mean ± standard deviation (SD) of three independent experiments. A *p*-value < 0.05 was considered statistically significant.

### UV Damage Protection Activity

HaCaT cells were seeded in 96-well plates at 5,000 cells/well and serum-starved for 24 h prior to UV exposure. After washing with PBS, cells were irradiated with solar UV (sUV, 30 kJ/m^2^) which is composed of 95% UVA (315-400 nm) and 5% UVB (280-315 nm) for 50 min in a lamp (Q-Lab Corporation, USA). Immediately after irradiation, cells were treated with distilled soju byproducts at concentrations of 100, 500, and 1,000 ppm and incubated for 24 or 36 h. Cell viability was assessed using the MTT assay by adding 10 μl of MTT solution (5 mg/ml) for 2 h, dissolving formazan crystals in 100 μl of DMSO, and measured absorbance at 570 nm. Results were expressed relative to non-UV exposed controls.

### Wound Healing Assay

HaCaT cells were seeded in 24-well plates at 1 × 10^5^ cells/well and cultured until confluent. A scratch was made using a sterile 200 μl pipette tip, and cells were rinsed with PBS to remove debris. Serum-free medium containing distilled soju byproducts (100–10,000 ppm) was added, and cells were incubated for 24 h. Images were taken at 0 and 24 h using an inverted microscope (Zeiss, Germany), and wound closure was quantified with ImageJ software as the percentage of the wound area.

### Statistical Analysis

All experiments were performed in triplicate, and data are presented as mean ± standard deviation (SD). Statistical analyses were conducted using GraphPad Prism (version 8, GraphPad Software Inc, USA). Data were analyzed using two-way ANOVA followed by Bonferroni’s multiple comparisons test to determine statistical significance between treatment groups. A *p*-value < 0.05 was considered statistically significant.

## Results

### Antioxidant Activity and Total Phenolic Content of Distilled Soju Byproducts

**Fig. 1:**
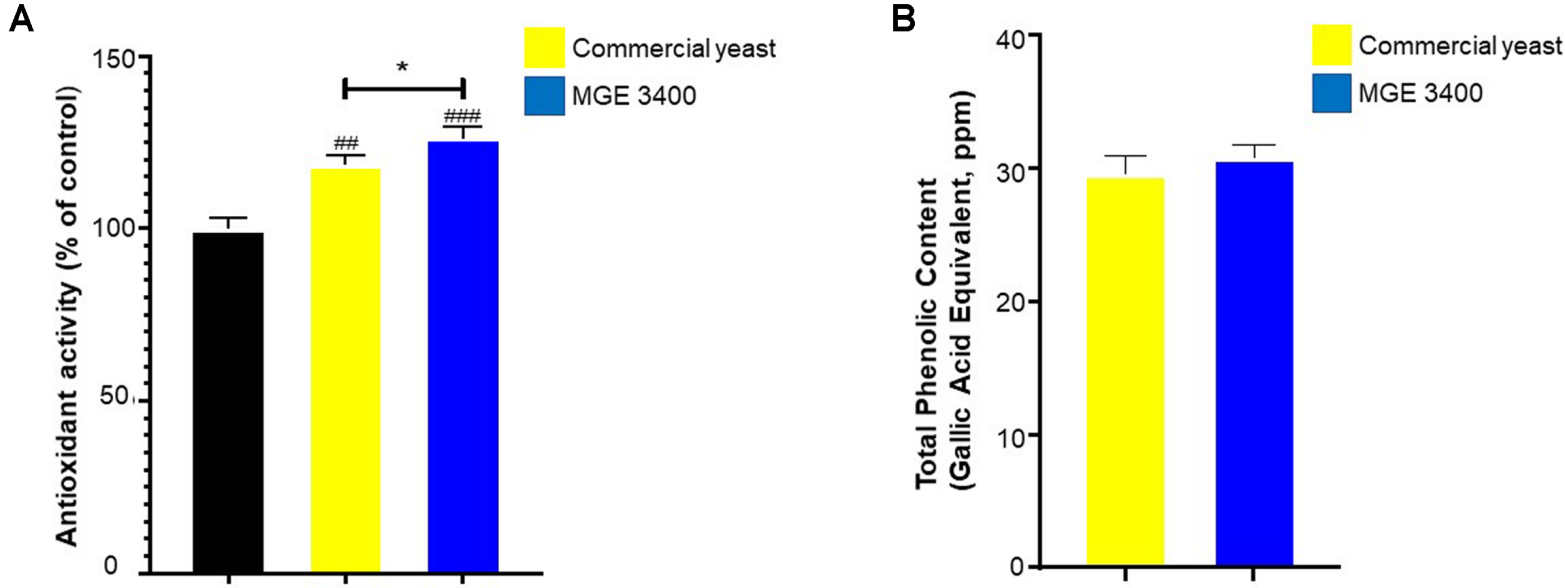
Antioxidant activity and total phenolic content of distilled soju byproducts fermented with commercial yeast or *S. cerevisiae* MGE 3400. (**A**) Antioxidant activity of distilled soju byproducts (DSBP) measured using the DPPH radical-scavenging assay. Both commercial yeast and MGE 3400 fermented samples showed significantly higher antioxidant activity compared with the untreated control, with the MGE 3400 exhibiting a further increase. (**B**) Total phenolic content of DSBP determined using the Folin-Ciocalteu method and expressed as gallic acid equivalents (ppm). The MGE 3400 fermented byproducts showed a slightly higher phenolic content than the commercial yeast fermented samples. Data are presented as mean ± SD (*n* = 3). Significance markers: ## *p* < 0.01 and ### *p* < 0.001 vs. control; *p < 0.05 between the two yeast strains.

The antioxidant activity of DSBP fermented with commercial yeast and *S. cerevisiae* MGE 3400 was assessed using the DPPH radical scavenging assay (Fig. 1A). Both fermented samples showed enhanced antioxidant capacity compared with the untreated control. Notably, the MGE 3400 fermented sample exhibited significantly greater radical-scavenging activity than the commercial yeast group (*p < 0.05), indicating that the MGE 3400 strain more effectively enhances the antioxidant potential of the byproduct. In addition, total phenolic content was measured using the Folin–Ciocalteu method (Fig. 1B). The MGE 3400 fermented byproducts showed higher TPC than the commercial yeast group. These findings indicate that phenolic compounds may contribute to the antioxidant activity of the DSBP. The superior activity observed in the MGE 3400 fermented DSBP may also involve additional antioxidant metabolites produced during fermentation.

### Antimicrobial Activity of Distilled Soju Byproducts

**Fig. 2:**
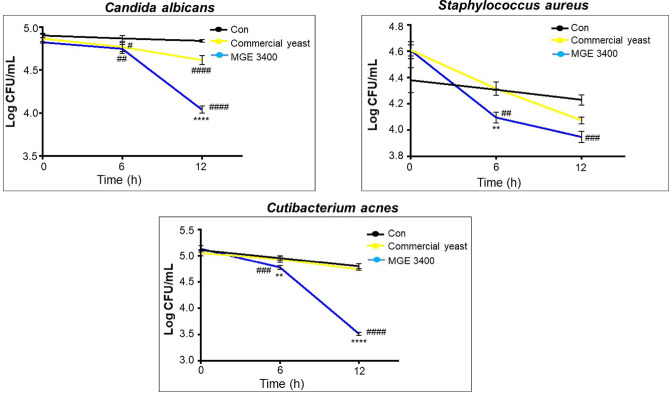
Antimicrobial activity of distilled soju Byproducts fermented with different yeast strains against *C. albicans*, *S. aureus*, and *C. acnes*. The antimicrobial activity was assessed by monitoring viable cell counts (log CFU/ml) over a 12-h period. Treatments included control, distilled soju byproducts fermented with commercial yeast, and *S. cerevisiae* MGE 3400. Data are presented as mean ± SD of three independent experiments (*n* = 3). Statistical significance between commercial yeast and MGE 3400 at the same concentration is indicated by ** and **** corresponding to *p* < 0.01, and *p* < 0.0001, respectively. Statistical differences compared with the untreated control within each group are indicated by #, ##, ### and #### corresponding to *p* < 0.05, *p* < 0.01, *p* < 0.001, and *p* < 0.0001, respectively.

The antimicrobial potential of the distilled soju byproducts prepared with commercial yeast and *S. cerevisiae* MGE 3400 was assessed against three microorganisms: *C. albicans*, *S. aureus*, and *C. acnes*. In the case of *C. albicans*, treatment with *S. cerevisiae* MGE 3400 significantly reduced the viable cell count compared with both the control and the commercial yeast group, showing a decrease from approximately 4.8 to 4.0 log CFU/ml at 12 h. For *S. aureus*, both commercial yeast and *S. cerevisiae* MGE 3400 exhibited stronger antimicrobial activity than the control, with *S. cerevisiae* MGE 3400 showing the greatest reduction in bacterial growth over time. The most striking effect was observed against *C. acnes*, where *S. cerevisiae* MGE 3400 markedly suppressed bacterial growth, reducing cell counts from around 5.2 to below 3.5 log CFU/ml, while the control and commercial yeast groups showed only slight decreases (Fig. 2). These findings indicate that distilled soju byproduct fermented with *S. cerevisiae* MGE 3400 possesses more potent antimicrobial activity compared with the control and commercial yeast.

### Effect of Distilled Soju Byproducts on HaCaT Cell Viability

**Fig. 3:**
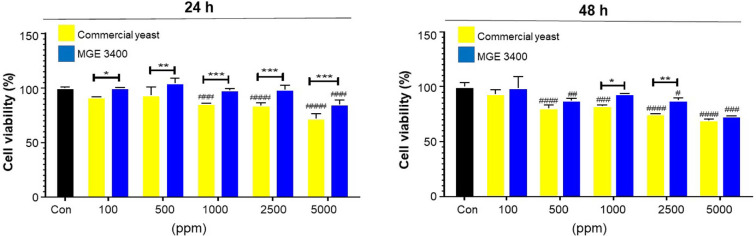
Effect of distilled soju byproducts fermented with commercial yeast and *S. cerevisiae* MGE 3400 on cell viability. HaCaT cells were treated with distilled soju byproducts fermented using either commercial yeast (yellow bars) or *S. cerevisiae* MGE 3400 (blue bars) at concentrations of 100–5,000 ppm for 24 h (left panel) and 48 h (right panel). Cell viability was measured using the MTT assay and expressed as a percentage relative to the untreated control. Data are presented as mean ± SD from three independent experiments (*n* = 3). Statistical significance between commercial yeast and MGE 3400 at the same concentration is indicated by *, ** and *** corresponding to *p* < 0.05, *p* < 0.01, and *p* < 0.001, respectively. Statistical differences compared with the untreated control within each group are indicated by #, ##, ### and #### corresponding to *p* < 0.05, *p* < 0.01, *p* < 0.001, and *p* < 0.0001, respectively.

The effect of distilled soju byproducts fermented with commercial yeast and *S. cerevisiae* MGE 3400 on HaCaT cell viability was assessed after 24 h and 48 h of treatment at concentrations ranging from 100 to 5,000 ppm (Fig. 3). Relative to the untreated control, both fermented byproducts maintained normal viability at low concentrations, while higher concentrations, particularly 5,000 ppm, resulted in reduced cell survival. At 24 h, cells treated with MGE 3400 fermented samples exhibited significantly higher viability than those treated with commercial yeast across all concentrations. At 48 h, this pattern persisted at selected concentrations, with MGE 3400 maintaining significantly greater viability at 1,000 ppm (*p < 0.05) and 2,500 ppm (**p < 0.01). Although viability decreased at the highest concentration in both groups and time points, MGE 3400 consistently preserved higher cell survival than commercial yeast. Overall, these results indicate that MGE 3400 fermentation enhances the protective effect of soju byproducts and better supports keratinocyte viability compared with commercial yeast.

### Effect of Distilled Soju Byproducts on HAS2 Expression in HaCaT Cells

**Fig. 4:**
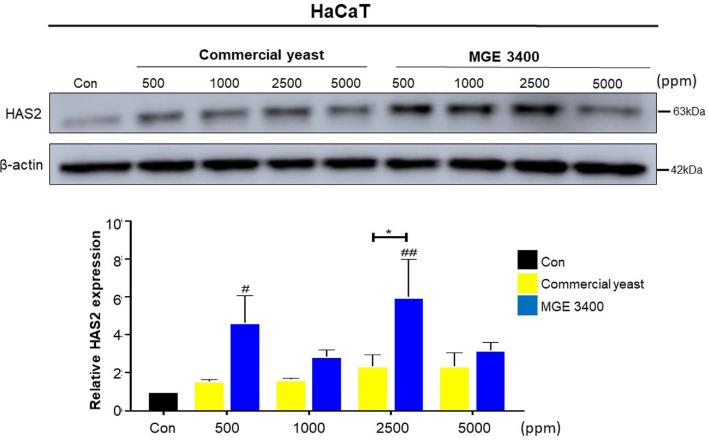
Effect of distilled soju byproducts on HAS2 expression in HaCaT keratinocytes. HaCaT cells were treated with distilled soju byproducts fermented with commercial yeast or *S. cerevisiae* MGE 3400 at concentrations of 500–5,000 ppm for 24 h. HAS2 protein expression was analyzed by western blot assay, with β-actin used as a loading control. Band intensities were quantified using ImageJ software (version 1.54 g, National Institutes of Health, USA), and relative HAS2 expression levels were normalized to β-actin. Data are presented as mean ± SD of two independent experiments (*n* = 2). Statistical significance between commercial yeast and MGE 3400 at the same concentration is indicated by * corresponding to *p* < 0.05. Statistical differences compared with the untreated control within each group are indicated by #, and ## corresponding to *p* < 0.05, and *p* < 0.01, respectively.

To evaluate the moisturizing potential of distilled soju byproducts, we examined the expression of HAS2 in HaCaT cells treated with various concentrations (500–5,000 ppm) of the byproducts fermented with either commercial yeast or *S. cerevisiae* MGE 3400 (Fig. 4). Western blot analysis showed that HAS2 protein expression was upregulated in both treatment groups compared with the control. However, the magnitude of induction differed between the two fermentation strains. Quantification revealed that MGE 3400 fermented byproducts induced a markedly stronger increase in HAS2 protein expression, with the highest response observed at 2,500 ppm (*p < 0.05). In contrast, commercial yeast byproducts produced only a modest increase across all concentrations tested. Collectively, these data indicate that MGE 3400 fermentation enhances the HAS2-stimulating activity of distilled soju byproducts more effectively than commercial yeast.

### UV Damage Protection Activity of Distilled Soju Byproducts

**Fig. 5:**
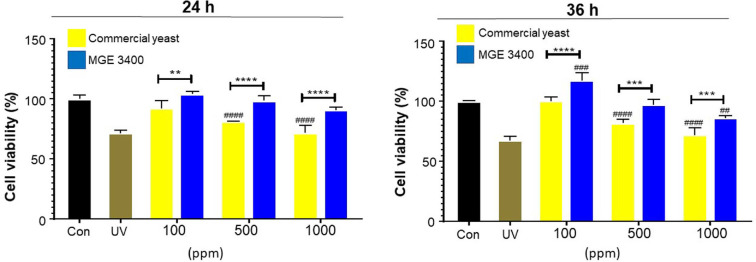
UV damage protection activity of distilled soju byproducts. Cell viability was measured after UV exposure in the presence of distilled soju byproducts fermented with either commercial yeast (yellow) or *Saccharomyces cerevisiae* MGE 3400 (blue). Cells were treated with 100, 500, or 1,000 ppm of byproduct or with 10% × PBS (UV–) and 10% × PBS (UV+) controls, and incubated for 24 h (left panel) or 36 h (right panel). Bars represent the mean ± SD from three independent experiments (*n* = 3). Statistical significance between commercial yeast and MGE 3400 at the same concentration is indicated by **, *** and **** corresponding to *p* < 0.01, *p* < 0.001 and *p* < 0.0001, respectively. Statistical differences compared with the untreated control within each group are indicated by ##, ### and #### corresponding to *p* < 0.01, and *p* < 0.001, *p* < 0.0001, respectively.

The UV damage protection activity of distilled soju byproducts fermented with *S. cerevisiae* MGE 3400 was evaluated at different concentrations (100, 500, and 1,000 ppm) and compared with commercial yeast at two time points (24 h and 36 h) (Fig. 5). UV irradiation significantly reduced cell viability compared with the untreated control. Treatment with both commercial yeast and MGE 3400 byproducts markedly recovered cell viability in a concentration-dependent manner; however, the magnitude of protection differed between the two fermentation strains.

At 24 h, treatment with *S. cerevisiae* MGE 3400 significantly increased protection against UV-induced damage compared with commercial yeast at all tested concentrations (100 ppm: **p < 0.01; 500 ppm and 1,000 ppm: ****p < 0.0001). After 36 h of treatment, the UV-protective effect of MGE 3400 became even more pronounced. Cell viability remained significantly higher than commercial-yeast treatment across all concentrations 100 ppm: ****p < 0.0001; 500 ppm and 1,000 ppm: ***p < 0.001).

These results indicate that *S. cerevisiae* MGE 3400 enhances UV damage protection more effectively than commercial yeast, and this effect is consistent across different concentrations and over time.

### Wound Healing Effect of Distilled Soju Byproducts

**Fig. 6:**
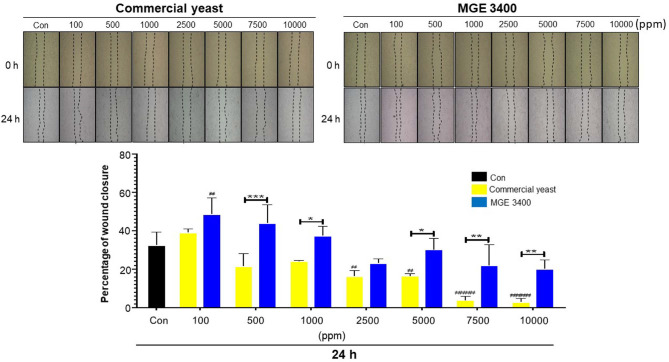
Effects of distilled soju byproducts on wound healing. HaCaT cells were treated with various concentrations (100–10,000 ppm) of distilled soju byproducts with commercial yeast or *S. cerevisiae* MGE 3400 for 24 h, and wound closure was monitored. Representative images of the scratch assay at 0 and 24 h are shown for each treatment group. The bar graph represents the percentage of wound closure at 24 h. Data are presented as mean ± SD (*n* = 3). Statistical significance between commercial yeast and MGE 3400 at the same concentration is indicated by *, ** and *** corresponding to *p* < 0.05, *p* < 0.01, and *p* < 0.001, respectively. Statistical differences compared with the untreated control within each group are indicated by #, and #### corresponding to *p* < 0.05, and *p* < 0.0001, respectively.

To evaluate the effects of distilled soju byproducts fermented with commercial yeast and *S. cerevisiae* MGE 3400 on wound healing, a scratch assay was performed. Results demonstrated that both groups exhibited concentration-dependent effects on wound closure (Fig. 6). At low concentrations (100–1,000 ppm), MGE 3400–fermented DSBP significantly enhanced wound closure compared with the untreated control, while commercial yeast–fermented DSBP did not show a comparable effect. MGE 3400 consistently produced a greater increase in keratinocyte migration than commercial yeast. The strongest wound-healing response was observed at 500 ppm MGE 3400 (***p < 0.001). While lower concentrations exhibited increased wound closure, higher concentrations (2,500-10,000 ppm) led to a progressive reduction in wound closure, suggesting cytotoxic or inhibitory effects at elevated doses. The enhanced closure suggests that fermentation with *S. cerevisiae* MGE 3400 produces byproducts components or alters the byproduct composition in a way that more effectively supports keratinocyte migration and wound repair.

### Effect of Distilled Soju Byproducts on IGF-1 Gene Expression

**Fig. 7:**
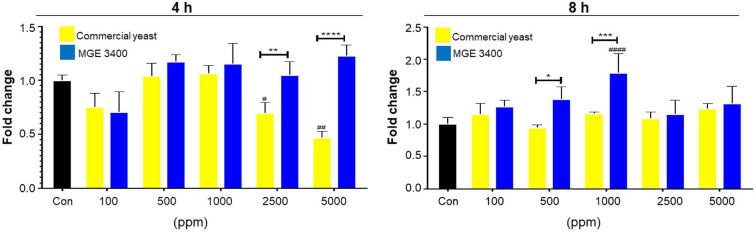
Comparison of IGF-1 gene expression between distilled soju byproducts with commercial yeast and *S. cerevisiae* MGE 3400. IGF-1 gene expression was measured after 4 h (left panel) and 8 h (right panel) of incubation at various concentrations of distilled soju byproducts (100–5,000 ppm). Yellow bars represent commercial yeast, and blue bars represent *S. cerevisiae* MGE 3400. Values are presented as mean ± SD from three independent experiments (*n* = 3). Statistical significance between commercial yeast and MGE 3400 at the same concentration is indicated by *, ** *** and **** corresponding to *p* < 0.05, *p* < 0.01, *p* < 0.001 and *p* < 0.0001, respectively. Statistical differences compared with the untreated control within each group are indicated by #, ## and #### corresponding to *p* < 0.05, *p* < 0.01and *p* < 0.0001, respectively.

To evaluate the effect of distilled soju byproducts on IGF-1 gene expression, HaCaT cells were treated with different concentrations (100–5000 ppm) of distilled byproducts prepared using either commercial yeast or *S. cerevisiae* MGE 3400, and IGF-1 mRNA levels were measured using qPCR at 4 h and 8 h. At 4 h, *S. cerevisiae* MGE 3400-treated groups showed a dose-dependent increase in IGF-1 gene expression, with a significant upregulation observed at 2500 ppm (**p < 0.01) and a more pronounced induction at 5000 ppm (****p < 0.0001) compared with commercial yeast. Similarly, IGF-1 gene expression remained elevated in *S. cerevisiae* MGE 3400-treated groups at 8 h point (Fig. 7). Significant differences between *S. cerevisiae* MGE 3400 and commercial yeast were detected at 500 ppm and 1000 ppm (*p < 0.05 and ***p < 0.001), with *S. cerevisiae* MGE 3400 maintaining higher IGF-1 gene expression levels. Collectively, these findings indicate that distilled soju byproducts fermented with *S. cerevisiae* MGE 3400 induces stronger IGF-1 gene activation than commercial yeast.

## Discussion

Fermentation byproducts have been considered important sources of bioactive metabolites with dermatological potential, and their composition depends largely on several factors such as the microbial strains used [28]. In this study, we investigated fermented distilled soju byproducts produced by the *S. cerevisiae* strain MGE 3400 and demonstrated their beneficial effects on multiple keratinocyte functions relevant to skin health, including cell viability, antimicrobial defense, UV protection, hydration, and wound repair. Instead of focusing on a single purified substance, we assessed the combined effects of metabolites derived from fermentation, which allowed polyphenols, fatty acids, organic acids, and other molecules to work synergistically to promote multi-pathway skin benefits.

Previous studies on residues from shochu, sake, wine, and beer indicate that fermentation generally produces polyphenols, organic acids, medium-chain fatty acids, esters, aldehydes, higher alcohols, and peptides, many of which exhibited antioxidant, moisturizing, antimicrobial, and pro-regenerative properties [21–23, 29]. Strain-dependent metabolic variation considerably affects the abundance and ratios of these compounds, thereby regulating their biological activities [30]. In this regard, the wider metabolite spectrum produced by *S. cerevisiae* MGE 3400 is probably responsible for its better performance than the commercial yeast–fermented byproducts.

Keratinocyte survival is indispensable to ensure the integrity, barrier function, and regenerative capacity of the skin [31]. However, aging and exogenous stressors induce mitochondrial dysfunction and reactive oxygen species (ROS) generation, impairing epidermal homeostasis. Yeast-fermented residues reportedly contain bioactive phenolic and flavonoid compounds, and fermentation with *S. cerevisiae* enhances their total phenolic content and antioxidant activity in the resulting byproducts [32, 34]. Consistent with these observations, the MGE 3400 fermented DSBP in our study showed higher phenolic content and significantly stronger antioxidant activity than the commercial yeast fermented DSBP (Fig. 1), indicating that this strain generates a richer antioxidant metabolite profile during fermentation. In addition to phenolics, yeast fermentation may produce small peptides and secondary metabolites that are cytocompatible and able to interact with skin-relevant pathways such as the production of collagen and hyaluronic acid [35]. These antioxidant-enriched metabolites likely contributed to the increased viability of HaCaT cells treated with the MGE 3400 fermented DSBP by minimizing intracellular ROS accumulation and maintaining cellular redox balance.

The antimicrobial findings further support the metabolic advantage of MGE 3400. *C. albicans*, *S. aureus*, and *C. acnes* are key opportunistic pathogens involved in fungal intertrigo, atopic dermatitis, and acne pathogenesis [36–38]. Antimicrobial strength is inherently strain-dependent due to the marked differences in the types and quantities of acids, peptides, and other extracellular metabolites produced by different yeast strains [39]. Polyphenols and organic acids can disrupt microbial membranes, increase permeability, collapse membrane potential, inhibit ATP synthesis, interfere with virulence pathways, and suppress biofilm formation [40–44]. Organic acids such as acetic, succinic, and pyruvic acids commonly released by *S. cerevisiae*, which inhibit microbial growth by diffusing into cells and inducing intracellular acidification and metabolic collapse [45–46]. Phenolic acids such as ferulic, caffeic, and p-coumaric acid further contribute to the growth inhibition through induction of oxidative stress and compromise of membrane integrity [47, 48]. In addition, *S. cerevisiae* release small antimicrobial peptides as a result of autolysis or proteolytic activity that can inhibit bacterial growth by disrupting microbial membranes [49]. Together, these mechanisms likely account for the stronger antimicrobial activity of MGE 3400 fermentation residues.

Maintenance of skin hydration is closely associated with extracellular hyaluronic acid, synthesized mainly by the enzyme hyaluronan synthase-2 [50]. HA regulates not only moisture retention but also keratinocyte and fibroblast signaling through CD44-mediated interactions with intracellular cytoskeletal and growth-factor pathways [51, 52]. In our study, treatment with MGE 3400 fermented DSBP significantly elevated the expression of HAS2, indicating enhanced HA synthesis and improved epidermal water-binding capacity. This effect is consistent with previous reports demonstrating that phenolic compounds such as ferulic acid can upregulate HAS2 and boost HA production in human dermal fibroblasts [53]. Similarly, another study showed flavonols, including kaempferol and quercetin dose-dependently increase HAS2 mRNA and augment HA secretion in human keratinocytes (HaCaT) after 24 h treatment [54]. The expression of HAS2 in keratinocytes is responsive to intracellular signaling through PKC, Ca^2+^/CaMKII, and MAPK pathways [55]. Thus, bioactive metabolites potentially present in MGE 3400 fermented DSBP could similarly activate these signaling pathways toward the enhancement of HAS2 expression, increased HA production, and hence improved epidermal hydration.

Ultraviolet radiation exposure can induce skin damage through DNA mutations, oxidative stress, and inflammation, all of which in turn contribute to the premature aging process and increase the risk of skin cancer over time [56]. It has been shown that fermentation generally enhances the antioxidant content in extracts, with improved UV protection and lowered production of ROS [57]. The phenolic compounds present in the winemaking byproducts include flavonoids, tannins, and anthocyanins, which have been previously reported to inhibit ROS production, prevent lipid peroxidation, and modulate endogenous antioxidant enzymes; hence they may function as natural UV filters, enhancing photo protection when combined with traditional sunscreening agents [58]. Phenolic acids, including caffeic and ferulic acid, have been documented to counteract UVA-induced oxidative stress in keratinocytes by supporting endogenous antioxidant systems and limiting MMP-1 upregulation [59]. Rosmarinic acid also exerts a protective effect on keratinocytes under environmental stress through the reduction of intracellular ROS accumulation, maintenance of mitochondrial function, and decrease in apoptosis [60]. Hence, the UV-protective ability of the MGE 3400 fermented DSBP is aligned with the generation of an antioxidant-enriched metabolite profile that occurred during the process of fermentation.

Keratinocyte migration is a crucial event during the process of re-epithelialization in wound repair [61]. Recent studies indicate that by-products from the wine industry enhance the migration of keratinocytes and, thus, the closure of wounds [62]. For instance, Carullo *et al*. demonstrated that grape seed extract caused a significant increase in wound closure in the scratch assay when compared with untreated controls [63]. Consistent with this, treatment with the MGE 3400 fermented DSBP significantly enhanced the rates of wound closure and also increased mRNA expression of IGF-1. IGF-1 is a well-recognized regulator of epidermal regeneration, promoting keratinocyte migration, enhancing re-epithelialization, stimulating hyaluronan synthesis, and supporting wound bed contraction [64]. Previous work has shown that some polyphenols, such as resveratrol and fisetin, directly upregulate IGF-1 in keratinocytes and trigger related β-catenin-linked regenerative pathways [65]. These compounds are commonly enriched or biotransformed during *S. cerevisiae* fermentation, suggesting that similar metabolites present in the MGE 3400 fermented DSBP might be responsible for the observed IGF-1 induction.

Taken together, the positive effects of distilled soju byproducts from MGE 3400 fermentation are likely due to strain-specific secretion of bioactive metabolites that support keratinocyte survival, hydration, antimicrobial activity, and wound repair. Although the precise chemical composition was not profiled in this study, the observed biological activities align with known functions of polyphenols, organic acids, and other fermentation-derived compounds. These findings highlight the potential of MGE 3400 fermentation residues as a renewable source of multifunctional cosmetic ingredients and encourage future work to identify active compounds, clarify their mechanisms, and optimize formulation strategies for dermatological applications.

## Conclusion

The present study demonstrates that distilled soju byproducts fermented with *S. cerevisiae* MGE 3400 has increased antioxidant, antimicrobial, moisturizing, UV-protective, and wound-healing activities compared with byproduct fermented by commercial yeast. These functional improvements are likely due to the strain-specific production of bioactive metabolites during fermentation. Importantly, these findings show the broader value of MGE 3400 for the sustainable use of byproducts. Large amounts of residues are produced during the manufacture of distilled soju, which are usually discarded. However, these materials fermented with a specific strain of yeast can be used to produce ingredients with dermatologically beneficial properties. This process contributes to the circular bioeconomy by minimizing waste and producing valuable products from an already established industry. With the demonstrated effects on HAS2 expression, IGF-1 induction, keratinocyte viability, and UV protection, MGE 3400-fermented byproducts have great potential to be natural and multifunctional cosmetic or dermatological active ingredients for applications such as moisturizers, anti-acne care, after-sun protection, and wound-recovery products. Future research should focus on the identification of the key active compounds and the evaluation of their long-term safety and efficacy in sophisticated formulations.
